# Animal models of PTSD: a challenge to be met

**DOI:** 10.1038/s41380-018-0272-5

**Published:** 2018-10-19

**Authors:** Gal Richter-Levin, Oliver Stork, Mathias V. Schmidt

**Affiliations:** 1 Sagol Department of Neurobiology, University of Haifa, Haifa, Israel; 2 The Integrated Brain and Behavior Research Center (IBBR), University of Haifa, Haifa, Israel; 3 Psychology Department, University of Haifa, Haifa, Israel; 4 Department of Genetics & Molecular Neurobiology, Institute of Biology, Otto-von-Guericke-University Magdeburg, Leipziger Str. 44, 39120 Magdeburg, Germany; 5 Center for Behavioral Brain Sciences, Universitätsplatz 2, 39106 Magdeburg, Germany; 6 Department of Stress Neurobiology and Neurogenetics, Max Planck Institute of Psychiatry, Munich, Germany

**Keywords:** Neuroscience, Drug discovery

## Abstract

Recent years have seen increased interest in psychopathologies related to trauma exposure. Specifically, there has been a growing awareness to posttraumatic stress disorder (PTSD) in part due to terrorism, climate change-associated natural disasters, the global refugee crisis, and increased violence in overpopulated urban areas. However, notwithstanding the increased awareness to the disorder, the increasing number of patients, and the devastating impact on the lives of patients and their families, the efficacy of available treatments remains limited and highly unsatisfactory. A major scientific effort is therefore devoted to unravel the neural mechanisms underlying PTSD with the aim of paving the way to developing novel or improved treatment approaches and drugs to treat PTSD. One of the major scientific tools used to gain insight into understanding physiological and neuronal mechanisms underlying diseases and for treatment development is the use of animal models of human diseases. While much progress has been made using these models in understanding mechanisms of conditioned fear and fear memory, the gained knowledge has not yet led to better treatment options for PTSD patients. This poor translational outcome has already led some scientists and pharmaceutical companies, who do not in general hold opinions against animal models, to propose that those models should be abandoned. Here, we critically examine aspects of animal models of PTSD that may have contributed to the relative lack of translatability, including the focus on the exposure to trauma, overlooking individual and sex differences, and the contribution of risk factors. Based on findings from recent years, we propose research-based modifications that we believe are required in order to overcome some of the shortcomings of previous practice. These modifications include the usage of animal models of PTSD which incorporate risk factors and of the behavioral profiling analysis of individuals in a sample. These modifications are aimed to address factors such as individual predisposition and resilience, thus taking into consideration the fact that only a fraction of individuals exposed to trauma develop PTSD. We suggest that with an appropriate shift of practice, animal models are not only a valuable tool to enhance our understanding of fear and memory processes, but could serve as effective platforms for understanding PTSD, for PTSD drug development and drug testing.

## Introduction

### Posttraumatic stress disorder (PTSD)

Since the early days of mankind, traumatic events have been known to lead to disabling responses [1], but only in 1980 was PTSD officially included as a diagnostic category in the Diagnostic and Statistical Manual of Mental Disorders-third edition (DSM-III). However, in recent years the increasing societal challenges have brought PTSD to the center of attention. Combat-related trauma and associated PTSD are of considerable relevance to military, but military personnel are certainly not the main population associated with the disorder. Climate changes have led to large-scale natural disasters, affecting civilian populations. The global refugee crisis has exposed millions of children, women, and men to danger and exploitation. Further, people living in relatively stable communities are increasingly exposed to work and car accidents, terror attacks, sexual and physical attacks, or domestic violence that may lead to PTSD. The 12-month prevalence of PTSD across the world is estimated to be of 3–4% [2], and its estimated prevalence in conflict-affected populations increases to over 15% [3]. Furthermore, PTSD is associated with comorbidity with depression or substance abuse [4], which exacerbates the outcome and complicates treatment. In fact, many authors consider PTSD a rather heterogeneous disorder which likely contains several subtypes, such as complex PTSD, with specific characteristics such as somatization, dissociation, and affect dysregulation [5] if not distinct sub-pathologies [6, 7].

While PTSD is now recognized as a major health challenge, there is as yet only partially effective treatment for the disorder. First-line treatments of PTSD are forms of cognitive therapy, mainly cognitive–behavioral therapy, cognitive therapy, and exposure therapy [8, 9]. While effective in many cases, nonresponse to psychological therapies for PTSD may be as high as 50% [10]. The situation with psychopharmacology treatment is probably even worse. Despite the fact that drugs, such as serotonin selective reuptake inhibitors (SSRIs), are often prescribed to PTSD patients, several meta-analysis studies suggest that in fact the efficacy of such treatments is equivalent to that of placebo treatment, and even in studies that ascribe some beneficial effects to such treatments, its efficacy is minor [11, 12]. Low efficacy of SSRIs is not specific to PTSD, as similar concern has also been raised regarding their efficacy in major depression [13]. The World Health Organization-supported meta-analysis study by Hoskins et al. [14] indicates that the effect sizes for pharmacological treatments for PTSD compared with placebo are low and inferior to those reported for psychological treatments [14].

The low efficacy of psychopharmacological treatment of PTSD could have been expected to be translated to attempts to introduce novel drugs and drugs of novel mechanisms of action. However, the drugs in use are in principle not different from those used more than 40 years ago [15], and there seems to be an agreement that meaningful advancements with regard to the pharmacotherapy of PTSD will likely come only from the identification of mechanistically novel agents [13]. Despite the obvious need for better PTSD drugs, leading pharmaceutical companies have abandoned psychiatry drug-discovery programs, since those are considered high-risk activity. A critical factor contributing to this outcome is the poor (or too one-sided) understanding of the neural mechanisms underlying PTSD and the uncertainty of whether animal models of trauma exposure and fear memory are sufficient to predict a positive treatment outcome with a sufficient level of certainty [16, 17].

On the other hand, there is intensive academic research aiming at elucidating the neural mechanisms underlying PTSD, both in patients and in animal models. A recent review indicates four brain functions which are considered to play a role in the psychopathology of PTSD, including emotion regulation and executive function, threat detection, contextual processing, and fear learning. The review brings evidence for the involvement of associated brain circuits which are suggested to be dysfunctional in PTSD patients [18]. These are brain regions that have long been indicated in PTSD, including the amygdala, the medial prefrontal cortex, the anterior cingulate cortex, and the hippocampus [18]. While these findings are of great importance to our understanding of the neural circuits related to PTSD, a more thorough cellular and molecular understanding of the mechanisms of PTSD is required in order to be translated to novel or improved pharmacological agents.

### Clinical characteristics of PTSD

In 2013, the American Psychiatric Association revised the PTSD diagnostic criteria in the fifth edition of its Diagnostic and Statistical Manual of Mental Disorders (DSM-5) [19]. The revised diagnostic criteria include:

A. Direct or indirect exposure to a traumatic event, with an emphasis on the extraordinary magnitude of the event. This emphasis and the implications to animal models of PTSD will be further discussed below.

B. Intrusive symptoms.

C. Avoidance behavior.

D. Negative alterations in cognitions and mood.

E. Alterations in arousal and reactivity.

F. Duration of symptoms of more than 1 month.

G. Functional significance.

H. As always in psychiatric diagnosis—symptoms are not due to medication, substance use, or other illness.

In addition, the DSM-5 introduced an additional subtype of PTSD for children ages 6 years and younger, with a cluster of symptoms adapted to those young ages.

When considering modeling PTSD in animals, there are clearly some symptoms, like intrusive thoughts, which are not possible or are difficult to measure. Nevertheless, in each set of criteria there are measures that can be carried out within the context of the animal model. For example, intense or prolonged distress after exposure to traumatic reminders or marked physiologic reactivity after exposure to trauma-related stimuli may be measured in rodents as part of assessing criterion B 'Intrusive symptoms'. Likewise, avoidance behavior in face of trauma-related external reminders may be measured for criterion C 'Avoidance behavior'. Criterion D, of negative alterations in cognitions and mood, may be assessed by several, well-established tests such as tests of hedonic preference, social preference, and motivation. Measuring symptoms associated with criterion E, relating to alterations in arousal and reactivity, are of course quite straightforward to measure in animals, since there are many validated tests for irritability or aggression, hypervigilance, startle reaction, and sleep parameters.

It is also important to pay attention to the fact that according to criterion F, relevant symptoms are those that last for more than 1 month. It is not clear if 1 month in human’s life is translated exactly to 1 month in rodent’s life, but clearly it is of importance to consider symptoms which last for a significant period of time after the exposure to the trauma.

Importantly, criterion G refers to the functional significance of the symptoms. This is an aspect which in animal models has not been given enough attention as yet. Probably the most important implication of the symptoms above for patients is the resultant impairment of social and occupational functioning. Identifying a clear functional impairment associated with those symptoms of PTSD which can be measured in animals will be significant in securing the validity of the definition of an animal as clearly being affected.

In parallel to the criteria listed in DSM-5, more symptom-based stratifications of psychiatric disorders have been proposed, most prominently the Research Domain Criteria (RDoC) concept framed by the US National Institute of Mental Health (NIMH) [20]. In contrast to the disease classification and disease-specific diagnostic criteria of the DSM-5, RDoC defines 5 domains or constructs of observable behavior and neurobiological measures that have common underlying neurobiological circuits. These domains defined so far include (1) negative valence, (2) positive valence, (3) cognitive processes, (4) social processes, and (5) arousal [21]. Each of these domains can consequently be analyzed on different levels, ranging from genes and molecules over cells and neural circuits up to physiology and behaviors. As both the research domains and the levels of analysis are highly translational between humans and animal models, the RDoC concept also holds immense promise with regard to PTSD research. Of the defined five general domains, at least four are altered in PTSD patients (negative valence, positive valence, cognitive processes, and arousal), and these can be modeled in animals. Implementing the RDoC concept in clinical practice as well as translational research will take time, though, and many aspects of this concept are still heavily debated. For example, additional domains that are affected in PTSD patients (among others) have been proposed, such as stress and emotional regulation [22]. Nonetheless, when considering to model PTSD in animals it is highly useful to take the RDoC concept into account and align the model with the research domains and levels of analysis described herein.

### Genetics of PTSD: mechanistic insights from patient studies

Twin studies have demonstrated a heritability of PTSD risk of up to 30–40%, indicating the contribution to genetic risk factors to the disorder [23]. When considering that many genetic risk factors for PTSD are likely modulated by environmental influences via gene × environment interactions, the actual genetic contribution to PTSD may be even higher. Unfortunately, similar to the situation for many other psychiatric disorders, such as major depression [24] (often comorbid to PTSD), the use of unbiased genome-wide association studies to identify novel candidate genes has so far only been of limited success [25, 26]. Ongoing efforts with larger sample size may yet reveal reproducible hits, but the effect size of single polymorphisms is likely to be very small. Moderately better success has been obtained by investigating candidate genes, especially when the studies were focusing on gene × environment interactions.

The involvement of the stress system, especially the hypothalamic–pituitary–adrenal (HPA) axis is well documented for PTSD [27]. Multiple studies reported a hypoactive HPA axis in PTSD patients, related especially to a hypersensitive glucocorticoid receptor (GR) which is directly responsible for negative feedback regulation of the HPA axis [28]. Consequently, polymorphisms in genes involved in negative feedback regulation of HPA axis activity have been identified to be significantly associated with PTSD [29]. Next to described risk polymorphisms in the glucocorticoid receptor gene itself, the GR co-chaperone FKBP51 (encoded by the FKBP5 gene) emerged as a very interesting PTSD candidate gene. The best understood function of FKBP51 is the reduction of GR sensitivity. Importantly, FKBP5 is one of the most GR-regulated genes in the body, thereby forming an ultra-short feedback loop via mediation of GR sensitivity [30]. Polymorphisms in the regulatory region of the FKBP5 gene affect the GR-dependent transcriptional regulation of FKBP51 and consequently the sensitivity of the GR [31, 32]. The same polymorphism has been associated to interact with early-life trauma to predict adult PTSD [33–36], providing strong clinical evidence for a role of FKBP51 in moderating the risk for PTSD dependent on early-life experiences. While specific drugs acting on FKBP51 have now been developed [37, 38], a proof of principle that those drugs act in animal models of PTSD is still missing.

As learning processes and fear memory are central in PTSD pathology, it is not surprising that neural plasticity genes have also been implemented in this disorder, especially brain-derived neurotrophic factor (BDNF). BDNF is crucial for neural plasticity, as it promotes cellular growth and synaptic changes, and is also regulated via the GR. A polymorphism in the human BDNF gene, the so-called Val66Met polymorphism, gives rise to a functional BDNF variant. Carriers of the Met allele are considerably more frequent among PTSD patients compared to controls and homozygous Met carriers are at higher risk to suffer from PTSD [39]. However, BDNF has been linked to a variety of other psychiatric disorders, especially mood disorders [40], and hence it is yet unclear how specific the BDNF effects are in relation to PTSD symptomatology.

A third example for candidate-driven studies for PTSD is genes involved in monoaminergic signaling in the brain. Several studies have reported increased PTSD risks related to serotonergic or dopaminergic transporters [41, 42]. On the other hand, several studies and meta-analyses failed to replicate these associations [43], again indicating that the effect sizes of single polymorphisms are generally low and likely need to be studied using more complex gene × environment interactions. Overall, it is clear that the lack of a better understanding of the cellular and molecular mechanisms associated with PTSD hampers a better understanding and ultimately a more effective treatment of the disorder.

### Translational insights from established PTSD animal models

The main tool allowing detailed investigation into cellular and molecular mechanisms associated with a disease is the employment of animal models, which enable the resolution required for such studies. Animal models for understanding the neurobiology of PTSD are expected to unravel the cellular and molecular mechanisms associated with PTSD, which should serve to reveal novel targets for drug development. Furthermore, such models are also required as a platform for novel drug testing.

The fact that, in contrast to other psychiatric disorders, PTSD onset is associated with a clear triggering event—the exposure to a trauma—gave rise to hopes that this disorder will be relatively straightforward to model, and thus lead to relatively rapid progress in the understanding of PTSD neurobiology and the development of effective novel drugs [44, 45].

A good example for the successful use of established PTSD animal models in elucidating the involvement of a clinical candidate gene in PTSD pathology is BDNF [46]. Differences in BDNF expression levels have been reported for a number of PTSD animal models, although there is no clear picture regarding the direction of change [47–49]. Interestingly, mice carrying the human Met allele were shown to display delayed extinction learning which is in line with the human genetic associations and suggests a causal link to PTSD pathology [50]. Furthermore, BDNF infused into the infralimbic medial prefrontal cortex reduces conditioned fear for up to 48 h, suggesting that boosting BDNF activity in certain brain circuits may be used to treat PTSD symptoms [47]. With the development of small-molecule mimetics for the tropomyosin receptor kinase B (TrkB) receptor [51], potential new treatment options for PTSD arise. However, the differential and widespread role of BDNF in various brain regions makes this a difficult target for pharmacological intervention.

The situation is similar for molecular insights in HPA axis functioning and interventions from animal models of PTSD. Several studies confirmed GR-dependent signaling abnormalities in PTSD animal models. For example, Daskalakis et al. [52] identified GR signaling as the convergent pathway associated with individual differences in a rat model of PTSD in both males and females. Further, the GR co-chaperone FKBP51 is dynamically regulated in fear extinction models and linked to GR agonist-dependent enhanced fear extinction [53], thereby supporting the human FKBP51-related genetic findings. The first selective FKBP51 antagonist has recently been developed [37] and shown to reduce anxiety in mice [38], underlining the need for further studies with these compounds in PTSD models.

There are more examples of increased mechanistic insights gained from established PTSD animal models [54] (see Table 1). Furthermore, animal models have started to examine the possibility of device-based treatments for PTSD, such as deep-brain stimulation (DBS). However, despite intensive research (e.g., reviewed in refs. [55–58]), the expectation that the in-depth understanding of the mechanistic underpinnings of the available PTSD animal models, and the hope for identifying novel and more effective drugs based on these insights, remain unfulfilled as yet [15].

**Table 1:** Factors with genetic PTSD/PTSD feature association in human and corresponding findings in animal models

Gene (human findings)	Finding in animal model(s)
**HPA stress axis**	
Glucocorticoid receptor [211, 212]	GR stimulation improves fear extinction after stress-enhanced fear conditioning [53] GR expression is increased in the PFC after single prolonged stress [213] Transcriptional changes of the GR pathway in amygdala and hippocampus after predator scent [52] GR mediates potentiation of fear memory after single prolonged stress [214]
FKBP5 [215, 216]	FKBP5 knockout prevents age-induced impairment of stress resilience [217] FKBP5 knockdown in the rat infralimbic cortex enhances extinction [218]
… *Shows interaction with juvenile adversity on PTSD development* [33, 36]	*Reduced FKBP5 in expression in the rat PFC after early-life stres*s [219] *Lastingly increased FKBP5 expression in rat BLA after chronic mild stress in adolescences* [220]
CRH/CRHR [221–223]	Conditional ablation of CRHR1 from forebrain neurons impairs consolidation of remote fear memory [224] CRHR2 mediates stress-enhanced fear conditioning via Mek1/2 activation [97] CRHR2 knockdown in BNST provides resilience in a stress-enhanced fear learning paradigm [225] CRHR2 overexpression in BNST attenuates predator stress-induced fear in susceptible animals [226]
… *Mediates the effect of juvenile adversity on cortisol response* [227]	*Transient prepubertal overexpesion of CRH in the forebrain increases vulnerability* [228] *Early-life stress* *×* *5-HTT interaction controls CRH Promoter methylation in adult* [229]
PACAP / PAC1R [230]	PAC1R−/− show reduced anxiety [231] PACAP HET produce increased vulnerability to combined juvenile and adult chronic mild stress [232]
**Serotonergic system**	
5-HT transporter [233–236]	5-HTT−/− mice show fear extinction deficits [237] 5-HTT inhibitor venlafaxine relieves forced swim stress after single prolonged stress [238]
… *Mediates interaction of childhood adversity and sex on hippocampal volume* [239]	*5-HTT mutation interacts with maternal separation stress to control HPA maturation and behavior in adult rats* [240, 241]
5-HT1A receptor [242]	5-HT1A knockout shows increased context fear memory [243] 5-HT1A mediates fear extinction deficits after early-life stress [100] Increased 5-HT1A expression in dorsal raphe after single prolonged stress [244] 5-HT1A mediates recovery of inhibitory control in the dentate gyrus after juvenile stress [245]
**Dopaminergic system**	
Dopamine receptor 2 [246]	Regulation of DRD2 expression in N. accumbens by prenatal stress [247] Reduced DRD2 expression in amygdala after social stress induction of increased fear [248]
Catechyl-*O*-methyltransferase [249]	—
… *Mediates lastingly increased cortisol levels in adolescents after stress* [250]	*COMT−/− mice are vulnerable to cannabinoid treatment in adolescence, altering PPI in adult* [251]
**Glutamatergic system**	
mGluR5 [252]	Mediates stress-enhanced fear memory through interaction with Homer [98]
**GABAergic system**	
Glutamic acid decarboxylase [249, 253]	GAD2 knockout shows increased, generalized fear [254] GAD2 knockout displays extinction deficits [255]
	*GAD2 haplodeficient mice show resilience to fear generalization after juvenile stress* [256]
GABAA receptor	Rescue of PTSD symptoms in single prolonged stress model by midazolam [257]
… *Interacts with childhood trauma towards risk for PTSD* [258]	*Altered GABAA receptor alpha subunit expression in juvenile stressed rats* [259] *Reduced allopregnanolone after social isolation and rescue of social isolation induced fear behavior by ganaxolone* [260, 261]
GABA-B receptor ... Only pharmacological evidence	Knockout of GABAB1a results in fear memory generalization [262] GABA-B antagonist induces fear generalization in mice [263] GABA-B antagonist blocks fear extinction in rats [264]
Cannabinoid receptor 1	Knockout mice show an increased response to repeated stress exposures [265] CB1 antagonist blocks conditioned fear extinction [266] Increased expression in dorsal striatum after a single prolonged stress [267]
… *Interacts with childhood abuse to increase fear in PTSD* [268]	*Disruption in adolescence alters adult anxiety-related behavior* [269]
**Neuropeptides**	
Neuropeptide Y [270]	NPY mediates resilience to effects of predator odor exposure [155] Reduced expression is observed following chronic variable stress shock [271] Inhibition in the dentate gyrus impairs context salience determination in fear conditioning [110]
Nociceptin/Orphanin FQ [272]	N/OFQ acts as anxiolytic following single prolonged stress in rats [273]
Tachikinin 2	Overexpression in central amygdala mediates consolidation of stress-enhanced fear in mice [274]
Brain-derived neurotrophic factor [246, 275–278]	Impaired fear extinction found in BDNF-e4 mutant mice [279] BDNF promoter methylation is increased in the hippocampus after psychosocial stress [280] Increased BDNF signaling is observed after single prolonged stress [49] BDNF cko show enhanced fear learning [281] BDNF knockdown in the hippocampus impairs fear extinction [282] Impaired fear extinction in BDNF Met-Val mutant mice [283]
… *Child abuse moderatesVal66Met induced increase of threat reactivity in PTSD veterans* [284]	*Critical for context fear memory in adolescent mice* [285]
Oxytocin/OT Receptor [286]	Reduced anxiety of OT−/− males mice [287] Increased anxiety in OT−/− females [288]
… *Mediates effect of early-life stress on adult depression anxiety stress scale* [289]	*OT−/− mice show reduced vocalization during maternal separation* [287]
**Others**	
Apolipoprotein E2 [290, 291]	APOE2−/− mice display lack of fear extinction [291, 292] APOE2−/− mice show increased response to chronic variable stress [292]
Interleukin-1 receptor [92]	IL-1−/− mice display increase in conditioned fear [89] Blockage of IL-1 in the hippocampus ameliorates stress-enhanced fear memory [95]
S100B, only serum levels	Knockout mice show increased conditioned fear [93] Increased CSF levels found after maternal stress+adult shock [94]
Regulator of G protein signaling 2 [91]	RGS2−/− mice show increased context fear memory [90]
Voltage gated calcium channel subunit alpha 1C [293]	Enhanced fear memory in CACNA1C+/− mutant mice [294]
ROR alpha [295]	ROR-A−/− mice show increased corticosterone response to novelty stress [296]
… *Mediates effect of early-life stress on posttraumatic stress response* [297]	*ROR-A promoter methylation upon maternal separation stress prevents differentiation of adult neural precursors* [298]

The italic fields indicate findings related to juvenile adversity

### The need for more effective animal models of PTSD

Developing an effective and translational animal model of PTSD is not a trivial task. As with other psychiatric disorders [59, 60], there are inherent challenges from both ends of the mission. PTSD is not a well-defined disorder. The definition and diagnosis of PTSD in humans is based on behavioral symptoms and self-reports, without any objective parameters, and there is a large overlap with other disorders, including mood disorders, anxiety disorders, as well as alcohol and drug abuse [61–63]. As a result, it is not clear what is required in an animal model in order for the model to reliably reflect the human disorder.

It is also not trivial to use the conventional ways to validate an animal model of PTSD. The main validation approaches are simply not possible [64]. Probably the strongest type of model validation is based on 'construct validation'. Construct validity points to the degree of similarity between the mechanisms underlying behavior in the model and those underlying the behavior in the condition which is being modeled [65]. While there are strongly based hypotheses regarding the neural mechanisms which underlie PTSD [18], the actual neural basis of PTSD is as yet not known, and thus it is not possible to validate the model relying on construct validity.

Likewise, may be the most common approach to validate an animal model of a disease is by pharmacological validation. It requires that a pharmacological treatment which reduces symptoms in humans will reduce the symptoms in the animal model with similar efficacy. However, there is no 'gold standard' pharmacological treatment in PTSD, and available treatments have low efficacy [11, 12].

## Towards an effective animal model of PTSD

The validity of existing PTSD animal models and their limitations have been reviewed before [58, 66–68], but a clear path for an improvement of the current situation is still under debate. The stalled progress in providing better understanding and improved treatments [15, 69] has been a major factor contributing to the withdrawal of leading pharmaceutical companies from psychiatry drug development [16, 17], but in addition, it raised serious doubts regarding the possibility of animal models to contribute to PTSD-related research and to related drug development (see, e.g., refs. [15, 69]).

Based on findings from recent years, we would like to propose a different view which does not ignore the progress so far but suggests that, with an appropriate shift of practice, animal models can become an even more valuable tool to enhance our understanding of the disorder, and could serve as effective platforms for drug development and drug testing. Towards that end, we here critically review various aspects of animal models of PTSD and propose research-based modifications that we believe are required in order to overcome some of the shortcomings of previous practice.

### When should the effects be measured?

Initially, the majority of individuals exposed to a severe trauma exhibit high-severity symptoms following the trauma that extinguish over time [70–72]. A similar temporal pattern is also found in animal models of PTSD [73]. There is much interest in studying the immediate or early responses to the trauma, both because those may hint to the way PTSD develops [74] and because it is assumed that, with better understanding of the neural mechanisms of the onset of PTSD, there could be an early window of opportunity for a potential effective intervention that could prevent the development of PTSD [75]. However, the majority of physiological, structural, and molecular changes observed after a trauma are likely to be adaptive and pro-resilience. PTSD is not defined by the immediate responses to the exposure to the trauma, but is rather a disorder of the lingering symptoms which fail to extinguish. According to the diagnosis criteria in humans, the same symptoms are considered as indicative of PTSD only after 1 month [19]. Accordingly, some researchers focus on behavioral, neurobiological, and physiological alterations which are found long after the exposure to the trauma [76–78].

Clearly, both the early effects of exposure to trauma and its long-term effects should be studied. In addition, the possibility of insidious effects, which develop over time, has been suggested, and should be further explored [76, 79]. It is important, however, to distinguish between findings obtained at different stages after trauma exposure, since those are likely to represent different aspects of the neurobiology of the emergence of PTSD.

### Modeling the trauma

PTSD is associated with the exposure to a significant trauma. This association has led many to consider the trauma as the cause of PTSD. This view, which will be challenged later on (see below and refs. [72, 73, 76, 80]), assumes that exposure to a sufficiently severe trauma would lead to the development of PTSD. The translation of this conception has led in many animal models of PTSD to a focus on the exposure to the trauma. In fact, many models are defined by the type of trauma the animals are exposed to (see Table 2).

**Table 2:** Commonly employed animal models of PTSD

Model	General protocol	Aims	Rationale	Reference number
Fear conditioning	A brief series of tone-shock exposures. Subsequent fear response to the tone, often in the form of freezing response, is measured as an index of pathology.	To model PTSD-associated enhanced fear response in face of reminders of the original trauma.	Long-term behavioral symptoms associated with exposure to a significant stressor.	[83]
Exposure to a short session of inescapable footshocks	Exposure to a brief (15 min) session of inescapable footshocks (e.g., 10 × 6 s, 1 mA) inducing a gradually developing and long-lasting change in behavioral responses to novel environmental stimuli.	To model an exposure to a significant, unescapable stressor.	An exposure to a significant stressor under uncontrollable conditions, which are assumed to enhance the aversive impact of the stressor. The protocol is expected to induce long-lasting mood symptoms.	[299]
Stress-enhanced fear learning (SEFL)	Pre-exposure to repeated footshock in one context produces an enhancement of conditional freezing to cues associated with a single shock in a second distinct context.	To model the history of stress exposure on the lasting effects of a traumatic event.	A high level of previous stress exposure enhances the risk for the development of PTSD symptoms.	[111]
Exposure to predators or predator odor	Involuntary exposure to a predator (cat, snake,..) or predator odor, often in combination with other stressors	To model a highly naturalistic stress situation for rodents.	Exposure to predators is considered an etiologically highly relevant stressor with a high likelihood to be perceived as a traumatic event.	[117, 152]
Single prolonged stress	Exposure to 2 h restraint, 20 min forced swimming and ether until loss of consciousness	Enhancement of lasting PTSD-like symptoms by combination of severe and systematically different stressors.	In order to induce a sufficient trauma in a rodent, the stress exposure needs to go beyond a naturalistic range of stressors and activate multiple brain circuits	[112]
Underwater trauma	Forced underwater submersion of the rat for 45 s	Creation of a highly traumatic and unescapable situation.	Exposure to this etiologically relevant and presumably highly traumatic event results in lasting PTSD-like symptoms, which can also be enhanced by later exposure to reminder cues	[114]
Immobilization/restraint	Single prolonged immobilization for 2 h with all 4 limbs on a wooden board, or prolonged restraint in a tube.	Creation of an inescapable situation and a severe psychological stressor with as a consequence long-term behavioral and neuroendocrine alterations.	Especially under laboratory housing conditions, where animals have little exposure to stressors, prolonged immobilization is a highly standardized and effective traumatic experience	[113]
Chronic stress models (e.g., chronic variable stress, chronic social defeat, etc)	Exposure of rats or mice to less severe, but chronic or repeated stress situations	Modeling a more prolonged stress exposure as risk factor for PTSD, potentially also the comorbidity with depression	Also chronic stress exposure is a risk factor for PTSD, and rodent chronic stress models result in PTSD-like phenotypes	[300]

#### The case of classical fear conditioning

Probably the earliest example was the use of classical fear conditioning. Fear conditioning is induced by exposure to a stressful stimulus, it has long-term impact, the onset of which follows the exposure to the stress, and it is closely associated with the amygdala, a brain structure clearly implicated in PTSD [81]. Indeed, Pavlovian fear conditioning paradigms have been repeatedly proposed to provide important insights into PTSD mechanisms [82, 83]. Undeniably, these have been instrumental in identifying brain regions and local circuits, transmitter systems, and a plethora of molecular factors that are required for or modulate fear learning. As may be expected, these include the typical factors involved in neuronal plasticity, including the mediators of glutamatergic, GABAergic, and neuromodulatory transmission, structural components and modifiers that control the dynamic reorganization of the cytoskeleton, as well as cell–cell and cell–matrix interactions, and various intracellular signaling pathways and regulators of gene expression. A number of excellent reviews are available that has summarized these findings (see, e.g., refs. [84, 85]). Moreover, the involvement of epigenetic modifiers lastingly controlling gene expression has gained increasing attention [86, 87].

The hypothesis behind the employment of fear conditioning paradigms as a model of PTSD was that PTSD is induced by an abnormally strong conditioned fear response. However, while it is clear that fear is abnormally regulated in PTSD, it is not clear whether individuals with PTSD acquire abnormally stronger conditioned fear responses [88]. Classical fear conditioning is a learning process which is normal and critical for animal survival. It is important to note that, when concluding from results of animal experiments using classical fear conditioning regarding potential mechanisms of PTSD, there is in fact an assumption in this approach that the mechanisms underlying PTSD are similar in principle to those of classical fear conditioning, only more intense. While this assumption could be correct, there is also the possibility that PTSD is a result of the collapse of the normal fear responses in the face of severe trauma, and the development of an alternative, pathological process.

Although fear conditioning may not as such be a model of PTSD, its investigation has been informative in identifying molecular and circuitry mechanisms which may be speculated to contribute to the biological base of PTSD. The aim of rodent PTSD models, however, must also be to generate predictions and to identify new potential entry sites for therapy. This is much more difficult to achieve using classical conditioning protocols as we need to cautiously differentiate between the processes that underlie adaptive fear memory formation and those that ultimately lead to pathology. In that respect gene mutations that result in exaggerated fear memories in mice, which are much less frequently observed than those disturbing fear memory formation, may be of interest. Two examples are the null mutant mice of the receptor for interleukin-1 [89] and of the regulator of G-protein signaling-2 [90], and both are indeed recognized as genetic factors in PTSD [91, 92]. Another such example is the glial protein S100B, ablation of which results in increased long-term potentiation and fear memory [93]. While a direct genetic link of S100B to PTSD is still missing, its level was found to increase in the cerebrospinal fluid (CSF) of mice following a combination of maternal separation stress and inescapable footshock in adulthood [94]. In fact, an increasing number of rodent studies is beginning to consider the effect of juvenile adversity, stress sensitization, and other PTSD risk factors on fear memory formation. Thus, the roles of interleukin-1 [95], MR [96], CRF2R [97], or mGluR5 [98] as mediators of stress-enhanced fear learning have been demonstrated. Moreover, early-life stress has been shown to impair conditioned fear extinction via changes in *N*-methyl-d-aspartate receptor–extracellular signal-regulated kinase signaling [99] and 5-HT1A receptor signaling [100] in the limbic system.

On the other hand, we have previously reported protective gene function of GAD65 haplodeficiency in juvenile stress-induced enhancement of contextual fear memory, although the full mutation recapitulates several PTSD features including generalization and lack of extinction after fear conditioning [101]. This illustrates the often non-linear nature of genotype–phenotype relations in these models and the specificity of phenotypic features. A promising approach for tackling these issues is certainly to target the molecular mechanisms involved in specific aspects of PTSD using opto- and pharmacogenetic tools, as done for fear memory incubation [102], fear memory generalization [103, 104], or conditioned fear extinction [105, 106]. Currently, opto- and chemogenetic studies mostly focus on the circuitry identification, but viral knockdown methods have also been introduced in order to dissect the molecular pathways involved in the GABAergic control and synaptic mechanisms of extinction [107–109], or the control of context memory salience [110] in fear conditioning models. The systematic application of these approaches to the critical features of PTSD will shed a new light on the involved processes, and should ultimately be fed into improved and individualized animal models.

#### Models of the trauma

Because PTSD is so closely associated with the exposure to a traumatic event, many of the current animal models of PTSD emphasize the type of trauma employed. For example, stress-enhanced fear learning (SEFL) [111] is a rodent model of sensitized responding to threat, in which exposure to a 15-shock stressor non-associatively enhances subsequent fear conditioning training with only a single trial. In another model, single-prolonged stress, proposed by Liberzon et al. [112], animals are exposed to restraint for 2 h, followed by forced swim for 20 min, followed by ether anesthesia. This protocol aims to mimic the assumed massive surge of cortisol, resulting from the exposure to the traumatic event, which is hypothesized to trigger the disorder. Restraint stress or immobilization stress is sometimes used by itself as a model of exposure to stress [113]. Other models of a single exposure to severe stress, which attempted to put emphasis on introducing etiologically relevant stressors, are the ‘underwater trauma’ model [114], sometimes also termed ‘submersion stress' [115], and ‘predator/predator odor exposure’ [115–117]. Still etiologically relevant but of longer exposure is the model of ‘social defeat' [118], in which animals are repeatedly exposed to a social defeat situation over several days.

In all these and similar models, the focus is on the type of trauma the animals are exposed to, under the assumption that, when exposed to a sufficiently severe trauma, animals will develop PTSD-like pathology. There seems to be no need to focus on a single type of exposure, because in humans, markedly different types of trauma are associated with the development of PTSD. It is, however, suggested that different types of trauma may lead to differences in the resultant subtype of PTSD [7]. A variety of animal models with exposure to different kinds of trauma should thus be useful to model the richness of the disorder but attention should be paid to possible differences in outcome between different types of trauma.

However, there are two important points that should be taken into consideration in respect to the choice of the trauma:

(A) The challenge of ethical considerations. There is no question that animal experimentations should be carried out thoughtfully, and should include ethical considerations. One key ethical principle is to reduce unnecessary suffering of the animals. However, another important principle is to perform experiments in a thoughtful way that would maximize the probability of gaining significant novel findings.

An animal model of PTSD presents an ethical challenge: an effective model is required to involve exposure to a significant trauma, sufficiently significant that it would model the real-life situations associated with PTSD. Emphasizing the principle of reducing suffering leads in some cases to minimizing the level or type of exposure, such that the other principle, that of maximizing the probability of gaining significant novel findings, is compromised. Often researchers choose a stressor that would be more acceptable by the ethical committees, even though it is not certain that such a stressor is really beyond the coping abilities of the animals [119]. Thus, one of the challenges on the way to establish effective animal models of PTSD is to work in cooperation with the ethical committees in order to ensure that the type and severity of the exposure is not compromised such that the relevance of the experiment to PTSD is conceded.

(B) The trauma is not a sufficient condition to induce PTSD. Probably one of the most important aspects to consider in an animal model of PTSD is the fact that most people exposed to trauma do not actually develop PTSD. While in western populations the life-time prevalence of severely stressful events is as high as 75–80%, only about 10% of this population will suffer from clinically relevant PTSD [120–122]. This proportion of affected versus non-affected individuals within the exposed population indicates that, in contrast to our intuitive thinking, the exposure to the traumatic experience may be a necessary but not a sufficient condition to induce the disorder. There must be additional factors that determine the outcome of the exposure to the trauma. The fact that a relatively low proportion of exposed individuals eventually develop PTSD is thus a critical factor that should be taken into consideration in animal models of PTSD. There are two ways, which are not mutually exclusive, to incorporate this factor in the animal models: first, to include in the model exposure to *risk factors* in addition to the exposure to the trauma, and second, to include in the model an individual profiling analysis that would enable identifying the affected individuals within the exposed population (*behavioral profiling*).

### The role of risk factors in animal models of PTSD

An important question in understanding the neurobiology of PTSD is why some individuals develop PTSD, while others exposed to the same trauma do not. Clearly, some form of a priori susceptibility should be assumed for a trauma to lead to PTSD. Indeed, individual behavioral traits are suggested to predict the response to trauma.

For example, it was found that long-term stress-induced sensitization of behavioral responsivity and somatic pain sensitivity could be predicted by low or high open-field locomotor reactivity [123]. Similarly, physiologic symptoms of analgesia, cognitive deficits, and hyporesponsivity of the HPA axis similar to those observed in human subjects with PTSD were demonstrated in an animal model of congenital learned helpless behavior [124]. High-trait anxiety, which was found to be associated with altered HPA axis activity [125], with differences in mineralocorticoid receptor (MR) expression in the hippocampus and with hippocampus functioning [126], was found to be associated with increased sensitivity to exposure to stress and to increased risk of developing psychopathologies [127]. In accordance with those findings, we could demonstrate that enhanced pretrauma anxiety in a fear-anhedonic and not anxious-only phenotype could predict the progression of posttraumatic anhedonia in a rat model of PTSD [76].

Accordingly, to understand the neural mechanisms of PTSD it is also important to study the mechanisms of such risk factors. Several risk factors have been suggested which may be categorized into background factors, distal life experiences, and proximal factors.

The main suggested background factors involve transgenerational epigenetic effects and genetic background [128–132]. Genetic background though is not sufficient to explain individual differences in sensitivity to stress and trauma, since individual differences are also found within genetically homogeneous populations, such as inbred mice (see, e.g., ref. [133]).

Distal life experiences may interact with a certain genetic background or may be influential enough to produce long-lasting alterations in coping abilities later in life. It can be expected that, since from birth to adulthood the brain is going through significant developmental alterations, early-life adversities would have somewhat different impact if occurring at different developmental stages. Studies should and do differentiate between interventions at different developmental stages, but the impact of distal life adversities has been reported for early-life adversities [134, 135], adversities during childhood [136–139], and during adolescence [140]. Those early-life adversities are suggested to induce alterations in expression profile of critical molecules but also to result in experience-induced epigenetic alterations [141] which can have long-term effects into adulthood [142]. Suggested proximal factors include for example drug and alcohol abuse, sleep deprivation, and illness [143, 144].

Risk factors and the mechanisms by which they hamper the ability to cope with trauma later in life should be studied to facilitate development of treatments that would reduce the risk of developing PTSD (see, e.g., refs. [78, 145]). In addition, risk factors should be considered to be included as part of the model of PTSD, both because they reflect the human condition and because they increase the proportion of affected individuals in the sample, which is a clear advantage when aiming to unravel the neurobiological processes involved in psychopathological symptoms. Importantly, exposure to challenges could sometimes build resilience. For example, moderate exposure to early-life adversity has been suggested to increase stress and trauma resilience in adulthood [146, 147] and experimental evidence for this is accumulating in both humans [148] and animal models [149–151].

### Behavioral profiling analysis as a critical element of animal models of PTSD

The individual variability in response to a trauma is evident by the relatively low proportion of individuals who eventually develop PTSD [120–122]. With respect to animal models of PTSD, this individual variability means that referring to the group averaged result, as is typically the practice, would be very inaccurate. It should be expected that, as in humans, within the averaged group, there will be some individuals that, despite being exposed to the traumatic event, have not developed the disorder, or display an intermediate phenotype that could be indicative of an increased risk for trauma-related pathologies in the future. It is thus important to develop ways to differentiate between the affected, intermediate, and the non-affected individuals within the exposed group.

One of the first groups to consider this approach were Cohen et al. [152]. They based the dissociation between ‘maladaptive’ and ‘well-adaptive’ responses on the magnitude of the response to the trauma, and differentiating between two extremes using arbitrarily selected cut-off behavioral criteria (CBC) that were based on performance in two successive behavioral tests (elevated plus maze and acoustic startle response tests). Employing this approach, it was possible to demonstrate that, as in humans [72], the prevalence rates of maladaptive responses to trauma dropped over time from 90% in the acute phase to 25% enduring/maladaptive response on day 7, which remained constant over 30 days [73]. This temporal profile, which is consistent in humans and animals, brings up the issue of when the test should be set for the symptoms to be of relevance to PTSD, an issue that will be discussed below.

The CBC approach has been highly productive, yielding a series of findings (see, e.g., refs. [153–155]) which probably could not have been identified otherwise. However, the CBC approach differs from the way diagnosis is done in humans by focusing the analysis on exposed individuals and comparing the performance among them. In order to approximate the diagnosis procedure in humans more closely, we have developed a variation of the CBC, termed ‘behavioral profiling' [76, 77, 139, 156]. Behavioral profiling is based on referring to the performance of a control, non-exposed group as defining the norm. The performance of this group in a carefully selected test battery is first analyzed, and for each behavioral measurement cut-off values are defined in a way which leaves 85% of the control population within the norm values. Notably, several behavioral measurements are used. This is done prior to examining the exposed groups. Only after the cut-off values are defined, an analysis of all animals in all groups is carried out. Importantly, in order for an animal to be defined as affected, it has to fall out of the defined norm in several parameters, rather than just one (e.g., 4 out of 6 or 5 out of 8). This way, similarly to humans, control animals may have one or two parameters out of the norm, and still not be defined as affected.

It was important to demonstrate that differentiating animals to ‘affected’ and ‘non-affected’ based on the behavioral parameters in this analysis had functional significance. It was indeed demonstrated that in response to a reminder of the trauma, the map of brain activation was significantly different between animals that were exposed to the trauma and developed symptoms (exposed-affected) and those that were exposed to the same trauma but did not develop significant symptoms (exposed-non-affected) [76].

The importance of employing the behavioral profiling approach became clearly evident in another recent study, where we examined GABAergic changes related to PTSD, focusing on alterations in the expression of α subunits of the GABA_A_ receptor in several brain areas. Animals were exposed to trauma, and 4 weeks later were tested in a battery of tests which enabled dissociating the animals to affected and non-affected individuals. Examining group means, a significant elevation in the expression of the α1 subunit was found in the amygdala and hippocampus of the exposed group. Because on average the exposed group exhibited significantly higher levels of symptoms, it was tempting to conclude that the elevation in expression levels of the α1 subunit is associated with the pathology. Such a conclusion would have led to focusing efforts on reducing the expression of α1 as a protective measure. However, when reanalyzing the same data with the behavioral profiling approach, differentiating between affected and non-affected individuals within the exposed group, it was found that the elevation in expression levels of the α1 subunit stemmed from the exposed, non-affected individuals. This surprising finding indicates that the elevation in expression levels of α1 is associated with resilience rather than with the pathology [77].

It is advisable to combine the incorporation of risk factors and behavioral profiling analysis. Combining these approaches may help evaluating potential risk factors, both in terms of symptom severity and in terms of increasing the proportion of affected individuals within the trauma-exposed group. An increase in the proportion of affected individuals is suggested to be a more sensitive measure for the contribution of risk factors than the intensity of symptoms [77, 156].

### Sex differences

PTSD is considered to be more prevalent in women than in men [157–161] to the extent that some reviews refer to sex as a risk factor for PTSD [157, 162]. Differences between the way that the gonadal hormones testosterone or estrogen interact with the HPA axis or modulate hippocampal functioning, reported both in humans and in animal models, have been suggested as contributing to this bias [163–166].

Nevertheless, this notion is debatable, with some surveys failing to demonstrate such sex-related bias (see, e.g., refs [167–169]). It is not trivial to develop a conclusive picture, in part because the types of trauma males and females are more often exposed to are different, and because social stigma and support associated with different traumas are not similar for men and women [160, 170]. In addition, it may be that the prevalence of expression of certain symptoms is different between males and females. It has been suggested that the assumed sex difference in the prevalence of PTSD may be explained, at least in part, by the choice of symptoms used for the diagnosis of the disorder which may lead to females being more likely than males to meet current criteria for PTSD (see, e.g.,refs. [171–174]).

Animal models reflect this complexity of sex differences [170, 175–177]. Male and female rodents respond differently to stress and trauma [170, 175, 176], and to risk factors associated with the trauma, such as prepubertal, or juvenile pre-exposure to stress [156, 177, 178]. On the other hand, it is important to note that when presented with a relevant trauma and relevant risk factors, both males and females may develop PTSD-like symptoms [156, 179]. Nevertheless, as in humans, the main pathological symptoms presented by males may differ from those presented by females [156]. For example, in the study of Horovitz et al. [156], only males exhibited impairment in the two-way shuttle avoidance task, only females exhibited anhedonia, but both sexs exhibited reduced exploratory behavior. Using only one behavioral test would have inevitably led to a wrong conclusion regarding stress vulnerability and sex differences.

### Posttraumatic stress, posttraumatic depression, and comorbidity

Comorbidity of mood and anxiety disorders is the rule rather than the exception [180–183], and this is not different when referring specifically to PTSD [168, 184]. Whether it is only comorbidity or should PTSD, as suggested [185–187], be sub-divided to posttraumatic stress disorder and posttraumatic depression is still debatable. In any case, there is a growing understanding that PTSD, which used to be considered an anxiety disorder (DSM4) [188], is a more complex disorder, in which patients exhibit a mixture of symptoms of anxiety, mood, and cognition [19].

The behavioral profiling analysis approach can be adopted to assess anxiety, depressive, and cognitive symptoms. Exploiting this possibility reveals that similar complexity also exists in rodents [76, 156, 189–194]. For example, Tsoory et al. [190] demonstrated that, among the exposed-affected individuals, some exhibited more anxious symptoms, whereas others exhibited more depressive symptoms. Developing this further, Ritov et al. [76] demonstrated that such categorization of the exposed-affected individuals based on behavioral symptoms was associated with correlative differences in maps of neural activation, indicating that the behavioral profiling has functional implications which are reflected in neural processing [76].

The behavioral profiling approach also corresponds with the concern raised in recent years in psychiatry regarding the diagnostic criteria as determined by DSM categories [195, 196] in the sense that, as long as there is no 'biological signature' to a disorder, our current ability to define a symptom as representing anxiety, mood, motivation, or memory is limited. Referring to the performance of a control, non-exposed group as defining the norm does not require defining the deviation from the norm as an anxiety or mood symptom. It is an indication for an ‘abnormal’ behavior which awaits further classification based on physiological, neuronal, and pharmacological findings.

### Susceptibility and resilience in the face of trauma

The fact that exposure to trauma leads to PTSD only in some of the exposed individuals suggests that, while some are susceptible, others are somehow resilient to the impact of the trauma. Understanding the neurobiology of PTSD involves the understanding of mechanisms of susceptibility and resilience. Furthermore, the realization that resilience is a possibility has opened the way to the possibility of interventions aiming at prevention of PTSD and of treatments aiming at promoting resilience (see, e.g., refs. [145, 197]).

Exposure of animals to stress, and much more so, exposure to traumatic stress results in many epigenetic, gene expression, and biochemical alterations and resultant alterations in activity in many brain areas (see, e.g., refs. [76, 198–202]). Typically, such changes are assumed to be associated with the pathology. However, findings suggest that resilience is an active process in which the neurons make active efforts to cope with the challenge (see, e.g., refs. [76, 139, 155, 203, 204]). Differentiating between those alterations related to the pathology and those related to resilience is far from being a trivial challenge. Behavioral profiling, which enables differentiating between exposed-affected and exposed-non-affected individuals, is a valuable tool that may help differentiating between resilience-related and pathology-related targets [77]. Importantly, it would be feasible to apply treatments that either increase resilience in an individual or alternatively reduce susceptibility, and the underlying mechanisms could be very distinct. As treatment of individuals before any trauma exposure is not feasible, though, it remains to be seen if the identified resilience/susceptibility mechanisms can also be utilized for treatment interventions.

## Summary

**Fig. 1:**
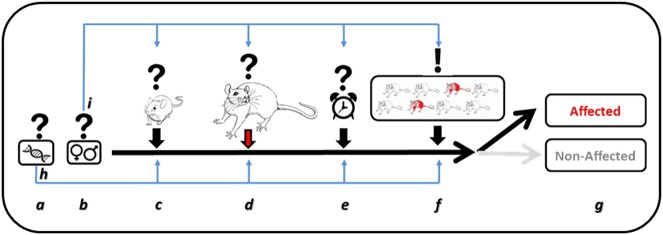
Guiding principles for selecting an animal model of PTSD. There is no single animal model of PTSD which as such is more adequate than others. Different models have their pros and cons. There are, however, several principles which are, in view of the authors, critical and should be incorporated in any model in order to increase the ecological validity of the models and their relevance to the human psychopathology. **a** Humans exposed to trauma are of heterogeneous genetic background. It should be natural to select outbred strains with a similar heterogeneous genetic background. Selecting an inbred line should be considered as a manipulation which defines the scope of the outcome of the study. As is indicated by the pale blue arrowed line (h), the genetic disposition may mediate effects at multiple levels, i.e., at the juvenile adversity, the trauma perception, and/or the individual outcome. **b** Males and females are sensitive to stress and trauma in different ways. The preference should be to examine both males and females. However, it should be borne in mind that specific manipulations may affect more either males or females. Likewise, males and females may differ in the behavioral aspects which are affected and thus different behavioral tests may be required in order to identify affected males or females. As is indicated by the pale blue arrowed line (i), sex differences may mediate effects at multiple levels, i.e., at the juvenile adversity, the trauma perception, and/or the individual outcome. **c** Most individuals exposed to trauma will not develop PTSD, indicating that the trauma will only be effective if it interacts with some additional pre-disposing factors. Studying the neurobiology of pre-disposing factors is thus a fundamental part of understanding the neurobiology of PTSD. Adding potential risk factors and examining their contribution should be considered, regardless of which trauma model is employed. **d** As is indicated in the text, there is no right or wrong with regards to which trauma should be employed in animal models of PTSD. Also, in humans different types of trauma may lead to the development of the disorder in some individuals. However, the choice of trauma and its parameters should be carefully considered and described, since this choice defines the relevance of the outcome of the study to exposure of similar nature in humans. Furthermore, researchers should address the question of the assumed severity of the traumatic experience. A clearer dissociation between stressful experiences (which are within the coping abilities of the animal) and traumatic experiences (which are beyond the coping abilities of the animal) is needed and should become part of the discussion of any study. **e** The age of exposure to trauma as well as the time after exposure for testing the impact of the exposure are important factors to consider. There is no right or wrong here but those choices define the relevance of the outcome of the study to exposure of similar nature and to the stage of evaluation in humans. **f** Because only some individuals exposed to a trauma will develop psychopathology it is critical to move away from analyzing the averages of the exposed and non-exposed groups. Instead, a more individual characterization of each animal as being pathologically affected or not is required. Towards that end, it seems important to aim for examining animals over batteries of tests that cover several behavioral faculties, in order to achieve a more reliable profiling of the individual animals. **g** Individual profiling of the animals could then be translated into defining individual animals as affected or non-affected, in a similar way to diagnosis in humans. With that type of analysis, effects of drugs can be examined as the impact of the proportion of affected/non-affected individuals, rather than on the averaged severity of specific symptoms

Recent years have seen growing criticism of animal models of psychiatric disorders in general and of animal models of PTSD in particular [15–17, 66, 69], up to the point of questioning the ability of such models to contribute to our understanding of the neurobiology of these disorders (e.g., ref. [15]). However, cumulative results from recent years indicate that animal models of PTSD are not only invaluable in understanding basic mechanisms of fear and memory processes, but are also able to capture the human complexity and differences of sensitivity to risk factors and to stressors, as well as the differences in the main resultant symptoms. However, in order for the models to be sensitive to such individual differences and to variations between sexes, the choice of risk factors, of the type of trauma, and of the behavioral measures of symptoms should be carefully selected before drawing conclusions. Importantly, moving the field towards a behavioral profiling analysis approach, which takes into consideration individual differences, is critical for being able to relate behavioral symptoms with their neural correlates and for establishing effective drug development and drug testing platforms. These novel developments in the way PTSD is modeled in animals, reviewed here, indicate that, with an appropriate shift of practice, animal models of PTSD are a valuable research tool for promoting our understanding of the neurobiology of PTSD (see Fig. 1 for summary).

Recently developed technology has further paved the way for this approach with (a) the rapid development of new chemo-optogenetic tools that allow us to target the intracellular localization activity of particular molecules as well as gene expression in defined cells and circuits [205, 206], (b) the evolution of CRISPR/Cas (Clustered Regularly Interspaced Short Palindromic Repeats/CRISPR-associated 9) technology which allows for the rapid generation of mutant mice and rats and base-specific engineering of any chosen gene in somatic target cells [207, 208] and single cell transcriptome analytics [209] providing unprecedented opportunities to examine the dynamics of activity and the molecular characteristics of such circuitries. Furthermore, the growing understanding of neural pathways involved in fear and PTSD is already suggested to be translated to new treatment approaches, such as DBS [210]. These methodologies should now be implemented in improved animal models of PTSD.
